# Modeling Parkinson’s Disease and Atypical Parkinsonian Syndromes Using Induced Pluripotent Stem Cells

**DOI:** 10.3390/ijms19123870

**Published:** 2018-12-04

**Authors:** Takayasu Mishima, Shinsuke Fujioka, Jiro Fukae, Junichi Yuasa-Kawada, Yoshio Tsuboi

**Affiliations:** Department of Neurology, Fukuoka University, Fukuoka 814-0180, Japan; mishima1006@fukuoka-u.ac.jp (T.M.); shinsuke@cis.fukuoka-u.ac.jp (S.F.); j-fukae@juntendo.ac.jp (J.F.); junkichi@marine.email.ne.jp (J.Y.-K.)

**Keywords:** induced pluripotent stem cells, Parkinson’s disease, atypical parkinsonian syndromes, multiple system atrophy, FTDP-17, Perry syndrome, proteinopathy, CRISPR

## Abstract

Parkinson’s disease (PD) and atypical parkinsonian syndromes are age-dependent multifactorial neurodegenerative diseases, which are clinically characterized by bradykinesia, tremor, muscle rigidity and postural instability. Although these diseases share several common clinical phenotypes, their pathophysiological aspects vary among the disease categories. Extensive animal-based approaches, as well as postmortem studies, have provided important insights into the disease mechanisms and potential therapeutic targets. However, the exact pathological mechanisms triggering such diseases still remain elusive. Furthermore, the effects of drugs observed in animal models are not always reproduced in human clinical trials. By using induced pluripotent stem cell (iPSC) technology, it has become possible to establish patient-specific iPSCs from their somatic cells and to effectively differentiate these iPSCs into different types of neurons, reproducing some key aspects of the disease phenotypes in vitro. In this review, we summarize recent findings from iPSC-based modeling of PD and several atypical parkinsonian syndromes including multiple system atrophy, frontotemporal dementia and parkinsonism linked to chromosome 17 and Perry syndrome. Furthermore, we discuss future challenges and prospects for modeling and understanding PD and atypical parkinsonian syndromes.

## 1. Introduction

### 1.1. Parkinson’s Disease and Atypical Parkinsonian Syndromes

Parkinson’s disease (PD) and atypical parkinsonian syndromes are age-related, progressive neurodegenerative diseases that are clinically characterized by parkinsonism including bradykinesia, tremor, muscle rigidity and postural instability. The most common type of parkinsonism is categorized as idiopathic PD (also designated as sporadic PD). PD is pathologically characterized by degeneration of dopaminergic (DA) neurons in the substantia nigra pars compacta (SNc) and by the presence of Lewy bodies, whose major components are aggregates of α-synuclein. On the other hand, familial PD has been considered to be a panel of genetic disorders whose patients have family histories of PD. A growing number of causative genes for familial PD have been identified since the first identification of α-synuclein (*SNCA*). Over past decades, a subset of susceptibility genes associated with PD have been discovered including *Leucine-Rich Repeat Kinase 2* (*LRRK2*), *glucocerebrosidase* (*GBA*), *SNCA* and *microtubule-associated protein tau* (*MAPT*) genes through genome-wide association studies (GWASs). In addition, a recent meta-analysis of the past GWASs identified more novel risk loci [1,2].

Atypical parkinsonian syndromes also exhibit parkinsonism due to loss of DA neurons caused by different pathological mechanisms. In addition, these diseases may accompany dementia associated with degeneration of cortical neurons. Atypical parkinsonian syndromes commonly include multiple system atrophy (MSA), progressive supranuclear palsy (PSP) and corticobasal degeneration (CBD). Relatively rare genetic diseases such as frontotemporal dementia and parkinsonism linked to chromosome 17 (FTDP-17) and Perry syndrome present parkinsonism, psychiatric features and cognitive decline. PD and atypical parkinsonian syndromes are classified into several types of proteinopathy [1,2,3,4,5,6,7,8,9,10,11,12]. In most cases, PD and MSA are pathologically linked to α-synucleinopathy, with the exceptions of *parkin RBR E3 ubiquitin protein ligase* (*PRKN*) and *LRRK2* [13]; PSP, CBD and FTDP-17 associated with *MAPT* gene mutations (FTDP-17 (*MAPT*)) are linked to tauopathy; Perry syndrome and FTDP-17 associated with *progranulin* (*GRN*) gene mutations (FTDP-17 (*GRN*)) are linked to TAR DNA-binding protein of 43 kDa (TDP-43) proteinopathy. However, it should be noted that these pathological features may frequently overlap each other. Numerous clinical and basic studies of these diseases have been performed but their pathological mechanisms remain elusive and no effective therapies to prevent neurodegeneration have been available so far. For the purposes of disease modeling, drug discovery and cell therapy, induced pluripotent stem cell (iPSC) technology has been expected as an important tool. This article focuses on iPSC-based models of PD and some representative atypical parkinsonian syndromes.

### 1.2. Brief History of Studies and Modeling of Neurodegenerative Diseases

Postmortem studies, especially clinicopathological studies on followed patients, coupled with family history studies, have greatly contributed to our understanding of the pathological aspects of neurodegenerative diseases and improvements in clinical diagnosis and eventually to the identification of causative genes [14]. Animal models provide important clues towards understanding disease mechanisms in vivo and the development of novel drugs; however, it has become clear that these drugs are not always effective in humans [15,16]. Indeed, many compounds that showed remarkable effects in animal models have failed in human clinical trials [17]. Such failures can occur because most mouse models do not faithfully recapitulate the full spectrum of human diseases [18]. For example, no obvious neurodegeneration of DA neurons has been found in almost all genetic mouse models of PD that were designed based on causative genes of familial PD [19,20]. Embryonic stem cells (ESCs) theoretically have the ability to differentiate into any cell type; however, in addition to solving immunological rejection in the host, there have been ethical issues, since human ESCs need to be obtained from fertilized human eggs [15]. Takahashi and Yamanaka established the technologies of mouse iPSCs [21] and then human iPSCs [22] from fibroblasts during 2006–2007. iPSCs can be derived from various somatic cells including fibroblasts and reprogrammed; they have similar characteristics to ESCs with regard to surface antigens, morphology, gene expression and differentiation potential [21,22,23,24]. Subsequent studies have revealed that human iPSCs are useful for human-specific disease modeling. In 2008, the first iPSC resources were established from patients with various types of genetic and multifactorial diseases, including PD and amyotrophic lateral sclerosis (ALS) [25,26]. ALS patient-derived iPSCs were differentiated into motor neurons [26]. The latter iPSC-ALS study [26] revealed that for ALS, the patient-derived iPSCs were successfully differentiated into motor neurons, raising the expectation of human cellular models being able to reproduce aspects of familial and sporadic neurodegenerative diseases [26]. Shortly after, iPSCs were generated from a spinal muscular atrophy (SMA) patient [27]. Importantly, the patient iPSC-derived neurons exhibited disease-related phenotypes and responsiveness to treatment, with drugs such as valproic acid, demonstrating that human iPSCs can be used for modeling of inherited diseases [27]. In 2009, for the first time, DA neurons were differentiated from iPSCs for PD patients [28]. Accumulative studies have been directed towards establishing cellular models of various human neurodegenerative diseases by using patient-derived iPSCs. So far, most of the studies have employed cellular models differentiated in vitro from patient-derived iPSCs, namely through indirect reprogramming strategies, rather than direct reprogramming; for example, from fibroblasts to neurons.

## 2. Cellular Modeling of PD and Atypical Parkinsonian Syndromes Using iPSCs

### 2.1. PD

PD is the second most common neurodegenerative disease after AD and patients with PD show both a variety of motor and non-motor symptoms. The non-motor symptoms include cognitive decline, mood disorders, sleep disturbances, sensory complaints, dysautonomia, executive dysfunction, autonomic dysfunction and gastrointestinal symptoms [29,30,31]. Motor symptoms of PD result from loss of DA neurons in the substantia nigra; therefore, most research efforts using PD-specific iPSCs have been focused on DA neurons. When PD patient-specific iPSCs were first generated in 2008, differentiation into DA neurons was not examined [25]. Shortly after, another group reported successful differentiation of iPSCs derived from idiopathic PD patients into DA neurons; however, no obvious phenotypes were detected in their idiopathic PD-iPSC-derived DA neurons [28]. PD is usually sporadic, with 5% to 10% of PD being familial [32] and so far, most of iPSC-based research on PD have focused on familial PD with confirmed genetic mutations. Most studied iPSC lines of familial PD have been carrying the G2019S mutation in the *LRRK2* gene, the most common mutation found in dominant familial PD [33]. The *LRRK2* G2019S homozygous mutation-harboring iPSC-derived DA neurons exhibited increased susceptibility to oxidative stress and caspase-3 activation and underwent cell death upon treatment with various stressors, such as the proteasome inhibitor MG132, hydrogen peroxide and 6-hydroxydopamine, as compared with control neurons [33].

Another study revealed that, following long-term culture, DA neurons derived from patients with idiopathic PD and *LRRK2* G2019S-mutated PD showed morphological changes in neurites, such as reduced numbers of neurites and neurite length [34]. Alterations in autophagic clearance were detected in the *LRRK2*-PD-iPSC-derived DA neurons by using LC3 as a marker of autophagosomes [34]. A systematic study, in which multiple laboratories conducted analyses in parallel, revealed that neurons differentiated from familial PD patient [*PTEN-induced putative kinase 1* (*PINK1*) Q456X homozygote, *LRRK2* G2019S homozygote, *LRRK2* R1441C heterozygote]-derived iPSCs exhibited increased vulnerability to various cytotoxins, such as the antibiotic valinomycin, the H^+^-ATPase inhibitor concanamycin A and hydrogen hyperoxide [35]. Live-cell imaging showed significant differences in mitochondrial dynamics and morphology between *LRRK2* G2019S or R1441C mutation-carrying iPSCs-derived neurons and control neurons. Vulnerability of iPSC-derived neurons from patients carrying *LRRK2* mutations to valinomycin and concanamycin A, which is caused by mitochondrial dysfunction, was rescued by pharmacological treatment with antioxidant coenzyme Q_10_, mTOR inhibitor rapamycin, or the LRRK2 inhibitor GW5074 [35]. Together, iPSC-derived neurons from PD patients have exhibited susceptibility to stressors causing cell death [34,36] and abnormality in mitochondrial dynamics and function [35], in addition to aberrant neuronal morphology in long-term culture [34].

The I2020T mutation, one of the first identified in the *LRRK2* gene, was also found in Japan [36]. I2020T mutant LRRK2 iPSC-derived neurons released lower levels of dopamine upon high KCl depolarization and showed vulnerability to oxidative stress and increased phosphorylated tau, which was caused by AKT/GSK-3β signaling abnormalities [36]. Consistently, the autopsied brain tissue of a patient from whom the iPSCs originated showed increased levels of phosphorylated tau and deposition of neurofibrillary tangles, thus establishing the *LRRK2* I2020 mutation-carrying iPSCs as a useful platform for studying PD pathology [36].

Furthermore, a targeted gene correction study in DA neurons derived from iPSCs of the *LRRK2* G2019S mutation-harboring patients demonstrated that their reproduced disease phenotypes, such as abnormality of neurite elongation, vulnerability to oxidative and mitochondrial stress and α-synuclein deposition, were ameliorated by genome editing using zinc-finger nucleases (ZFNs; see below) [37]. In addition, the authors showed that expression of several genes, the *CADPS2*, *CPNE8* and *UHRF2* genes, were dysregulated in the *LRRK2* G2019S mutation-carrying iPSC-derived neurons and their abnormal expression levels were dependent on the activity of extracellular-signal-regulated kinase 1/2 (ERK) [37].

Another representative gene for familial PD, the *SNCA* gene, has also been extensively studied using iPSCs. iPSCs were generated from an *SNCA* triplication patient and differentiated into DA neurons [38]. These neurons expressed α-synuclein protein at higher levels than control neurons established from an affected first-degree relative, raising the possibility that these model neurons are useful for identifying drug candidates capable of reducing α-synuclein levels. Another group also generated a human iPSC-based model derived from *SNCA* A53T mutation-carrying patients by directed differentiation [39]. The mutation-carrying iPSC-derived neurons showed the disease-relevant cellular phenotypes, such as protein aggregation, reduced neurite outgrowth, axonal neuropathological changes and synaptic abnormalities. Remarkably, in-silico-designed, small molecules targeting α-synuclein rescued the neuropathological phenotypes of the *SNCA* A53T-carrying neurons in this model. In addition, such drugs protected proteasomal inhibitor-treated neurons from apoptosis, reinforcing the effectiveness of iPSC-based cellular models of PD.

The *PINK1* gene encodes a mitochondria-targeted kinase involved in mitochondrial quality control. Mitochondrial depolarization-induced translocation of the Parkin to mitochondria was impaired in iPSC-derived DA neurons from the *PINK1* mutation-carrying PD patient [40]. Analyses of mitochondrial respiration and neural cell vulnerability to stressors, coupled with evaluation of drug effects, revealed that coenzyme Q_10_ and GW5074 rescued cell vulnerability in the *PINK1* mutation-harboring neurons to valinomycin and concanamycin [35]. In *PRKN* mutation-carrying iPSC-derived DA neurons, increased oxidative stress levels and the abnormality of dopamine release and uptake were detected and Parkin expression rescued such phenotypes [41,42]. Although it is relatively rare that *PRKN* patients bear Lewy bodies in the substantia nigra, α-synuclein accumulation was observed in the autopsied patient’s brain examined in that study. Strikingly, α-synuclein accumulation was reproduced within iPSC-derived neurons from the same patient [42]. A recent development of new quantitative assays for mitophagy highlighted such abnormalities in DA neurons from *PRKN* mutant iPSCs [43]. This technique will be useful for dissecting mitochondrial quality control mechanisms that work in differentiated DA neurons. Furthermore, a recent study revealed that *PRKN* or *PINK1* patient-derived DA neurons showed remarkable apoptotic tendencies and susceptibility to the mitochondrial stressor, rotenone [44]. The authors performed phenotypic screening of an FDA-approved drug library and found that the calcium antagonist benidipine effectively suppressed rotenone-induced apoptosis, showing that iPSC-based cellular models are practical platforms for drug screening [44].

While most recent studies focused their attention on DA neurons, Son et al. closely examined gastrointestinal symptoms and identified changes in gene expression profiles using intestinal organoids from the *LRRK2* G2019S patients-derived iPSCs [45]. Given this new direction, it is likely that PD-specific iPSC-based studies that examine both DA neurons and various other types of cells may gain momentum in the near future.

### 2.2. Multiple System Atrophy (MSA)

MSA is a sporadic neurodegenerative disease that clinically presents with dysautonomia with various combinations of parkinsonism, cerebellar symptoms and pyramidal signs. Clinically, MSA is divided into two types: MSA with predominant cerebellar ataxia (MSA-C) and MSA with predominant parkinsonian features (MSA-P). Pathologically, MSA is characterized by α-synuclein-positive glial cytoplasmic inclusion (GCI). Thus, MSA is classified into α-synucleinopathy; along with PD.

Oligodendrocytes were generated from iPSCs derived from patients with familial PD, MSA and healthy individuals [46]. Oligodendrocyte lineage progenitor cells and mature oligodendrocytes were obtained from iPSCs by using a modified method based on dual SMAD inhibition. This study revealed that oligodendrocytes were able to produce α-synuclein inside of their own cells during their maturation in vitro; however, the origin of aggregated α-synuclein found in oligodendrocytes of MSA patients still remains unclear. Further studies using iPSCs from more MSA patients will be needed to understand MSA pathogenesis. Because a library of iPSCs from PD and MSA including MSA-C and MSA-P patients was established [47], these iPSC models will provide a valuable resource for studying shared or specific mechanisms of different forms of α-synucleinopathies. In another recent systematic study, DA neurons were obtained from iPSC lines derived from patients with MSA-C and MSA-P [48]. In the differentiated neurons at 70 days in vitro (DIV), tau protein expression was drastically reduced in all the lines examined. In addition, a significant reduction of autophagic flow was detected, which may be linked to the onset of MSA [48].

### 2.3. FTDP-17

FTDP-17, a rare autosomal dominant neurodegenerative disease, is a form of early-onset dementia and parkinsonism [11,12]. FTDP-17 patients exhibit parkinsonism, personality and behavioral changes and cognitive impairment [11,12]. Genetically, FTDP-17 is classified according to mutations in the *MAPT (tau)* and *progranulin* (*GRN)* genes [11,12]. The FTDP-17 subtype associated with the *MAPT* mutation pathologically exhibits extensive tauopathy, while TDP-43–positive abnormal inclusions are the pathological hallmark in FTDP-17 associated with *GRN* mutations [11,12].

#### 2.3.1. FTDP-17 (*MAPT*)

The majority of *MAPT* mutations have been located around the microtubule binding domain, which may disrupt the ability of tau protein to bind to microtubules and cause dysfunction in intracellular trafficking. iPSCs obtained from patients carrying the *MAPT* N279K mutation were recently generated and differentiated into neural stem cells (NSCs). Such NSCs derived from patient-specific iPSCs exhibited impaired endocytic trafficking in a neuronal lineage-specific manner. Intracellular vesicular trafficking involving endosomes and multivesicular bodies and exosomes were evaluated using lipid raft marker, Flotillin-1. Elevation of Flotillin-1 levels was detected in FTDP-17 patient-derived NSCs. Flotillin-1 levels are also increased in the cortex of FTDP-17 patients with the N279K mutation, consistent with in vitro analysis in NSCs [49]. Interestingly, abnormal expression of EEA1 and LAMP1, markers of early endosomes and late endosomes/lysosomes, respectively, were also detected in NSCs from patient-specific iPSCs, suggesting defects in endolysosomal maturation in FTDP-17 (*MAPT*) patients.

In another study, iPSCs that were established from N279K and V337M mutant patients were differentiated into mature neurons including DA neurons [50]. The patient-specific iPSC-derived neurons showed increased fragmentation of tau protein and phosphorylated tau immunoreactivity and a reduction in neurite extension. A transcriptome analysis revealed remarkably altered profiles of gene expression in the patient iPSC-derived neurons. In an independent study using neurons differentiated from iPSCs derived from patients with N279K and P301L, the deficiency of mitochondrial transport and earlier maturation of electrophysiological activity were reported [51]. These neurons also recapitulated altered tau expression profiles in patients, showing abnormal, premature expression of the adult brain 4-repeat (4R) tau isoform.

iPSC-derived neurons, carrying either an intronic (IVS10 + 14C > T) or exonic (R406W) *MAPT* mutation, showed accumulation of misfolded tau and extracellular release, which was followed by neuronal death [52]. Accumulation and extracellular release of misfolded tau were effectively inhibited by pharmacological inhibition of neural activity and by using designer receptors exclusively activated by designer drugs (DREADDs) in *MAPT*-mutated neurons. These findings suggest that neural activity affects neurodegenerative processes. This report further indicated that the combination of an iPSC-based model with DREADDs to control neural activity may be applicable to other neurodegenerative diseases [52]. Interestingly, astrocytes derived from patient iPSCs of frontotemporal dementia (FTD) patients carrying the N279K mutation on the *MAPT* gene became larger and exhibited increased levels of *4R-tau* isoforms, elevated protein ubiquitination and increased vulnerability to oxidative stress [53]. In a recent study, genetic engineering was performed to introduce the IVS10 + 16 mutation into healthy donor-derived iPSCs [54]. Such healthy donor-derived, mutation-carrying neurons showed increased expression of 4R tau and this mutation-specific neurodegenerative phenotype of FTD. Thus, FTDP-17 (*MAPT*) model neurons have recapitulated some aspects of tauopathy in vitro.

#### 2.3.2. FTDP-17 (*GRN*)

Although the pathological mechanisms caused by *GRN* deficiency remain unclear, it has been shown that nonsense-mediated decay (NMD) of mutant progranulin mRNA contributes to haploinsufficiency [55]. iPSCs carrying the S116X mutation in the *GRN* gene were differentiated into neurons and microglia [56]. Such patient-specific iPSC-derived neurons showed increased sensitivity to cellular stress. iPSCs derived from another type of patients with the *GRN* (IVS1 + 5G > C) mutation were differentiated into cortical neurons [57]. Although the efficiency of differentiation for cortical neurons in patients deteriorated, this was improved by genetic correction via targeted genome editing. RNA-seq analysis suggested that the Wnt signaling pathway could serve as a therapeutic target for curing FTDP-17 (*GRN*) [57]. FTDP-17 patient-specific iPSC-based research focusing on both neurons and glial cells may open an avenue toward elucidating previously untapped mechanisms of neurodegeneration in this disease.

### 2.4. Perry Syndrome

Perry syndrome is a rare autosomal dominant neurodegenerative disease clinically associated with parkinsonism, depression/apathy, weight loss and respiratory symptoms. Histological findings showed severe DA neuronal loss in the substantia nigra and TDP-43 and dynactin abnormal inclusions in the neurons [9,58]. In 2009, *DCTN1* mutations in multiple families of patients with Perry syndrome were discovered [59]. The *DCTN1* gene encodes p150^Glued^, the largest subunit of the dynactin complex. p150^Glued^ is essential for cargo transport along microtubules, functioning with the dynein retrograde motor [60]. Eight point mutations have been identified: F52L, G71A, G67D, G71R, G71E, T72P, Q74P and Y78C in the *DCTN1* gene [58]. We recently proposed international diagnostic criteria for Perry syndrome and that the disorder should be termed ’Perry disease’ because we found that patients with Perry syndrome showed mostly uniform clinical, genetic and pathological features [58]. We then generated iPSCs from a patient with F52L and differentiated them into tyrosine hydroxylase (TH)-positive neurons. The resultant TH-positive neurons contained dynactin aggregates in the cytoplasm and recapitulated an aspect of the disease phenotype of Perry syndrome [61]. However, these neurons, unexpectedly, did not show TDP-43 aggregates. TDP-43 aggregates in Perry syndrome may be a secondary event and only become evident a long time after the formation of dynactin aggregates. In addition, patients with the F52L mutation show a clinical phenotype which is slightly different from that of patients with other *DCTN1* mutations; it generally appears with later disease onset and milder clinical symptoms compared to patients carrying the other *DCTN1* mutations. Therefore, a direct comparison of the characteristics in iPSC-derived neurons of this mutation and those of other *DCTN1* mutations may be necessary for a detailed understanding of the pathological mechanisms underlying Perry syndrome [61].

A reduction in TDP-43 protein levels improves neuronal activities in a Drosophila model of Perry syndrome [62]. It may be possible to treat patients with Perry syndrome by keeping appropriate levels of TDP-43. TDP-43 proteinopathy has also been implicated in ALS. Tracking an mRNA beacon in living cells showed impairment of axonal transport of TDP-43 cognate mRNA in motor neurons derived from ALS patients with the TDP-43 mutations [63]. By employing a similar approach, the axonal transport of cargo including TDP-43 granules should be examined in iPSC-based models of Perry syndrome. We summarize modeling of PD and atypical parkinsonian syndromes using iPSCs (Table 1).

## 3. Challenges and Prospects of iPSCs for PD and Atypical Parkinsonian Syndromes for Various Purposes

**Figure 1:**
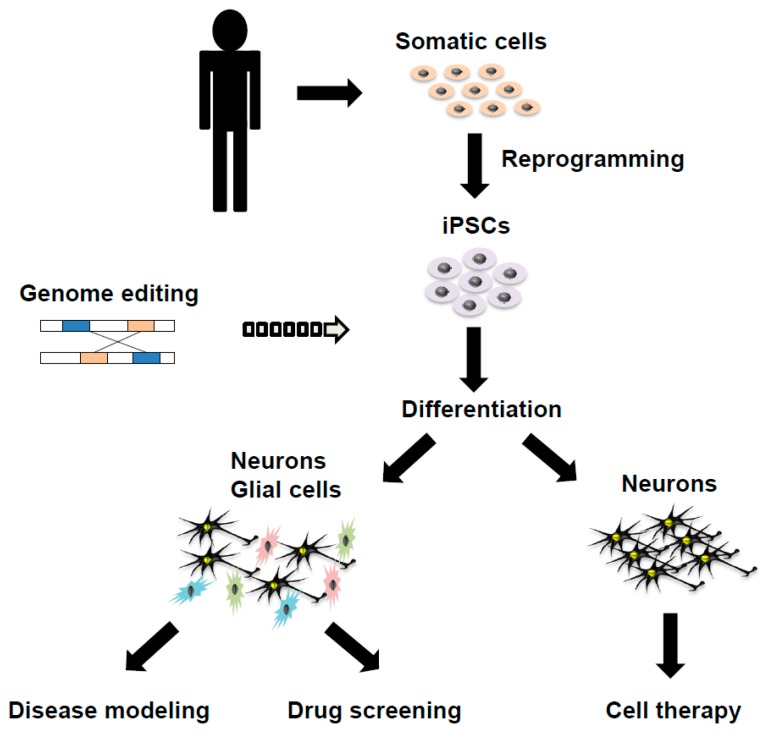
A schema showing the generation of disease-specific induced pluripotent stem cells (iPSCs) and its applications in disease modeling, drug screening and cell therapy on Parkinson’s disease and atypical parkinsonian syndromes.

Rapid advances in iPSC technology hold promise for its application in disease modeling, drug screening and cell therapy of various human diseases, including rare ones, many of which were not previously accessible for research purposes. iPSCs supply us with unlimited quantities of almost any type of differentiated cells, which recapitulate neurons and glial cells in patient brains if an appropriate cell differentiation protocol is established (Figure 1) [64]. We discuss some concerns about current approaches and techniques and future promising directions in iPSC technology for PD and atypical parkinsonian syndromes.

### 3.1. Improving the Methodology to Generate iPSCs and to Differentiate Them into Multiple Cell Types

iPSCs were initially generated via the integration of viral vectors encoding reprogramming factors into the genome and thus the risk of tumorigenicity has been pointed out in situations where iPSC-derived cells are transplanted into human bodies. To reduce such a risk in clinical applications, various integration-free techniques have been developed, for example, in the form of episomal DNAs, synthesized RNAs, or even recombinant proteins, and in combination with high-efficiency electroporation [15,16].

Many research groups have been generating iPSC lines using various methodologies, mostly state-of-the-art techniques at the time. One major problem is that collections of iPSCs could show significant variations, such as laboratory-to-laboratory, line-to-line (inter-individual or intra-individual variability) and even batch-to-batch (due to different efficacy of differentiation). The heterogeneity of differentiated neurons may compromise the reproducibility of an iPSC-based cellular model. Therefore, standardization of iPSC methodology is needed to ensure the reproducibility of obtained data and comparability of results. One solution may be the automation of reprogramming and differentiation steps for iPSC generation [65].

### 3.2. Cell Therapy Using iPSCs for PD and Atypical Parkinsonian Syndromes

“If cells could be derived from the patient’s own tissues, they would carry no risk of immunological rejection” [66]. iPSC technology has been expected to fit this purpose. Since the 1980s, fetal nigral transplantation in PD patients has been conducted; throughout the period of over 20 years since then, cases that have shown an improvement in symptoms and cell engraftment have been reported [67,68]. Despite such progress, ethical issues associated with the use of fetal tissues, the difficulty of obtaining sufficient amounts of fetal brain tissue to improve the symptoms and the occurrence of contamination of serotonergic neurons have posed a challenge to fetal nigral transplantation. Evidence of effectiveness in cell therapy alone is not enough to allow this procedure to become a widely accepted standard therapy. Given these dilemmas, cell therapy that takes advantage of iPSCs has recently generated growing hope in this area of research. Although improvements have been made in cell therapy, a number of issues need to be overcome before it can be applied to a clinical setting. Specifically, during the process of neural differentiation induction, there is a risk of tumor development when undifferentiated cells remain in the graft cells [69,70]. In order to reduce the risk, a cell-sorting technique has been developed utilizing an antibody against a floor plate marker, CORIN [71], a central nervous system microvascular endothelium marker, LRTM1 [72] and activated leukocyte cell adhesion molecule (ALCAM) [73]. Cell sorting not only improves the safety and the efficacy of the technique by enhancing the quality of graft cells but also maintains graft cell quality, thereby making it the most promising technique for clinical application. In fact, in a primate model of PD, human iPSC-derived dopaminergic progenitor cells survived and functioned as midbrain DA neurons [74]. Thus, cell therapy with iPSCs will hopefully be a promising approach for PD patients in the near future. In addition, mesenchymal stem cell therapy was able to delay the progression of neurological deficits in some cognitively intact MSA-C patients [75]. Therefore, further trials of cell therapy with iPSCs in both PD and MSA are warranted.

### 3.3. CRISPR-Based Genome Editing Technology in iPSCs

The advent of ZFNs and transcription activator-like effector nucleases (TALENs), enabled sequence-specific genome editing and the generation of isogenic controls as a strategy for providing a detailed analysis on hereditary diseases [58,76]. Following ZFNs and TALENs, the CRISPR/Cas9 system has been developed and widely used due to the simplicity and ease to use [77,78]. iPSC research coupled with the CRISPR/Cas9 system has recently attracted considerable attention.

The first report of bi-allelic targeted gene correction described the combination of ZFNs and *piggyBac* technology [79] and then the choice was switched to the CRISPR-*piggyBac* system. The refinement of the CRISPR system to introduce intended and flexible genomic correction in iPSCs is essential for establishing relevant disease model cells and isogenic control cells, in addition to applications to cell therapy. One of the future directions of research in this field will be on how to improve the accuracy of genome editing through homology-directed repair (HDR), although in CRISPR techniques, most double-stranded breaks (DSBs) are repaired by non-homologous end-joining (NHEJ), rather than HDR. A recent study reported a new CRISPR-based methodology, termed CORRECT, which enables accurate HDR-dependent introduction of intended mutations in a mono- or bi-allelic manner [80]. When we compare relatively small phenotypic differences between disease model cells and control cells, targeted gene correction allows us to establish genetically matched, isogenic control cells that are made using genome editing and to minimize effects of genetic backgrounds. The CORRECT technique not only eliminates differences in genetic backgrounds but also circumvents line-to-line variations of iPSCs. Rapid generation of genetically defined human iPSC-based disease models and corresponding appropriate control cells, using reference wild-type cells, is a strength of the CORRECT-based methods.

Although there have been many other technical improvements in CRISPR technology, there is still the need to reduce the risk of off-target effects for clinical applications and more refined disease modeling. Improvements of the nuclease Cas9 are also underway and new variants of Cas9, which exhibit reduced off-target effects, or which allow indel-free knockin in iPSCs, have been reported [81,82].

Collectively, iPSCs technology combined with CRISPR-based genome editing will continue to drive disease modeling of human neurological diseases, including PD.

### 3.4. Three-Dimensional Neural Culture

Although two-dimensional (2D) cell culture has predominated so far, the recent advancement in cell culture techniques and in vitro neural differentiation has enabled the development of three-dimensional (3D) organoid culture systems. Organoids recapitulate some aspects of in vivo developmental processes and mimic 3D tissue architecture through self-organization of cells. By using iPSC-derived differentiated cells, various forms of organoids have already been generated as new models of many human organs, including the brain, retina and kidney. In neurological research, the number of studies involving cerebral organoid models is likely to increase, because these reconstructed tissue structures are more similar to the actual cellular organizations and should reflect pathophysiological conditions more faithfully than 2D cultures [83,84]. As a PD model, *LRRK2* G2019S mutation-carrying patient-derived iPSCs were differentiated into 3D neuroectodermal organoids [45]. Microarray assays detected significant differences in gene expression related to synaptic transmission in the organoids between *LRRK2* G2019-carrying iPSCs and wild-type controls [45].

Techniques establishing region-specific 3D organoids of human brain, including forebrain and midbrain, have been developed rapidly [85,86]. In the organoids, neurons can undergo differentiation and further maturation through both extrinsic cues artificially added to cell culture and cell-cell interactions. For example, in the recently developed midbrain-like organoids, electrically active and mature midbrain DA (mDA) neurons were generated and dopamine production was detected [86]. Thus, modeling 3D architecture using multiple cell types differentiated from iPSCs will be essential for the next generation of disease modeling and may offer a new platform for drug screening, although it has already been studied, mainly aimed at organ/tissue replacement therapy. By using the organoids, it is possible to dissect cell-cell communication-based, namely non-cell-autonomous characteristics, in addition to cell-autonomous ones, of differentiated tissues and their disruption by neurodegenerative diseases.

However, one problem is that most 3D organoids only grow to small sizes, still far before mature organ development, possibly at least in part due to the lack of vascularization. The combined culture of brain organoids and vascular systems will be effective for promoting organoid growth and maturation. Furthermore, at the present stage, radial glia are not attached to the superficial pial surface and thus accurate lamination in the brain layer structures is not achieved [84]. In addition, the reproducibility of organoid formation is not still sufficient. However, undoubtedly, 3D brain culture technologies are promising tools for us to further understand brain function and to develop novel therapeutic strategies, by combining iPSC-based cell technology and CRISPR-based genome editing. Future disease modeling studies will also be focused on converting iPSCs into multiple neuronal subtypes with more physiologically relevant characteristics by stimulation with diverse extracellular ligands, including developmental patterning cues and small-molecule inhibitors in both 3D and 2D cell-culture systems [64].

### 3.5. Understanding Pathological Mechanisms of PD and Atypical Parkinsonian Syndromes by Using iPSC-Based Disease Model Cells

iPSC-based disease model cells have begun to act as effective platforms to uncover novel pathological mechanisms underlying PD. If it is possible to recapitulate the disease progression of PD and atypical parkinsonian syndromes more faithfully, this technique will progress toward understanding the pathogenesis and developing new therapeutic strategies. iPSC-based cellular models are also expected to contribute to the development of iPSC-independent therapeutic strategies, including small-molecule inhibitors to halt and/or retard disease progression, especially when coupled with diagnosis of early disease states using sensitive biomarkers. Thus, iPSCs will also become an essential tool for dissecting basic mechanistic research.

Genetic variations at the *SNCA* and *LRRK2* loci have been shown to confer a significant risk for idiopathic PD [87]. The gene encoding GBA, a lysosomal enzyme and causative factor of Gaucher disease, is also a key risk factor for PD [88]. *Vacuolar protein sorting 35* (*VPS35*), a major component of the retromer complex involved in endosomal trafficking, has recently been implicated in regulating mitochondrial dynamics [89]. Although evidence suggests that both mitochondrial dysfunction and endosomal/lysosomal dysfunction are involved in PD pathogenesis, how these physiopathological mechanisms are coupled with each other, and how their abnormal crosstalk causes PD, remain unknown. A recent study revealed that mitochondrial oxidative stress triggers the accumulation of oxidized dopamine in DA neurons derived from iPSCs from homozygous DJ-1 mutation-carrying PD patients and idiopathic PD [90]. DJ-1 has been implicated in regulating oxidant defenses [88]. Oxidized dopamine was shown to link mitochondrial dysfunction to lysosomal dysfunction, leading to α-synuclein accumulation. To further gain insights into how genetic and environmental factors, including stressors, contribute to the pathogenesis of idiopathic PD, iPSCs from individual patients will provide us with important in vitro cellular models.

### 3.6. iPSC Studies for Aging

To date, most studies involving patient-specific iPSCs have focused on inherited diseases and have aimed to reproduce the state of sporadic neurodegenerative diseases. Although some aspects of sporadic neurodegenerative diseases are closely related to aging, it is difficult to reproduce the state of aging in cell-culture studies and those concerning iPSCs are no exception. When iPSCs are established from somatic cells through reprogramming, their identity has to be reset. Thus, it is not easy to use iPSCs for cellular modeling of late-onset neurodegenerative diseases, including idiopathic PD. How to reproduce aspects of aging processes in vitro and in situ (such as organoids) is an important issue for improving cellular modeling of PD [91]. Sánchez-Danés et al. succeeded in phenotype reproduction using long-term culture after differentiation from sporadic PD patient-derived iPSCs to DA neurons [34]. Miller et al. examined Hutchinson-Gilford progeria syndrome (HGPS), which causes a rapid aging process and thereby attempted to reproduce late-onset types of PD through overexpression of progerin, a truncated form of lamin A, in DA neurons derived from iPSCs [92]. By accelerating the aging process of iPSC-derived neurons via progerin overexpression, it may be possible to improve the modeling of late-onset PD, especially idiopathic PD. Increased attention has been paid to progerin with the aim of advancing iPSC research that handles premature aging disorders including HGPS [93]; progression in this field of research may contribute to identifying factors accelerating research on clinical phenotypes in neurodegenerative diseases.

## 4. Conclusions

We have discussed the application of iPSC technology to establish cellular modeling in the research of PD and atypical parkinsonian syndromes. Since iPSCs entered the stage, there have been drastic improvements in accompanying, powerful methodologies of in vitro neural differentiation, genome editing and 3D organoid systems. iPSC technology will continue to be a key to solve shared and unique mechanisms underlying various neurodegenerative diseases, as iPSC-based studies of rare neurodegenerative diseases, other than Alzheimer disease (AD) and PD, have also been gradually accumulating. The ease of multiplying cell numbers is another strength of iPSC technology. If an appropriate procedure for cell differentiation and maintenance in vitro is established, large amounts of particular disease-model cells will become available for disease modeling, drug discovery and cell therapy. Although it is generally difficult to detect remarkable defects of DA neurons derived from idiopathic PD patient iPSCs, in vitro cellular aging procedures may enhance their disease-related phenotypes. Thus, the progress of iPSC-based technology and accompanying methodologies will push forward with more accurate modeling of neural cells, drug screening and also cell therapy to tackle such devastating neurological diseases.

## Figures and Tables

**Table 1:** Summary of modeling of Parkinson’s disease and atypical parkinsonian syndromes using induced pluripotent stem cells (iPSCs). PD, Parkinson’s disease; MSA, Multiple system atrophy; FTDP-17, frontotemporal dementia and parkinsonism linked to chromosome 17; *LRRK2*, *Leucine-Rich Repeat Kinase 2*; *PINK1*, *PTEN-induced putative kinase 1*; *MAPT*, *microtubule-associated protein tau*; TH, tyrosine hydroxylase.

Disease (No. of Patients)	Genetic Mutation	Cell Types	Phenotypes and Mechanisms	References
PD (*n* = 1)	*LRRK2* ^G2019S^	DA neurons	Increased susceptibility to oxidative stress and caspase-3 activation	[33] Nguyen et al., 2011
PD (*n* = 4)	*LRRK2* ^G2019S^	DA neurons	Morphological changes in neurites and impairment of autophagic clearance	[34] Sánchez-Danés et al., 2011
PD (*n* = 1)	*LRRK2* ^G2019S^	Neurons	Oxidative stress and mitochondrial dysfunction	[35] Cooper et al., 2012
PD (*n* = 1)	*LRRK2* ^R1441C^	Neurons	Oxidative stress and mitochondrial dysfunction	[35] Cooper et al., 2012
PD (*n* = 1)	*LRRK2* ^I2020T^	Neurons	Activation of GSK-3β and increased tau phosphorylation	[36] Ohta et al., 2015
PD (*n* = 2)	*LRRK2* ^G2019S^	DA neurons	Rescuing phenotypes by genome editing	[37] Reinhardt et al., 2013
PD (*n* = 4)	*LRRK2* ^G2019S^	Intestinal organoids	Changes in gene expression	[45] Son et al., 2017
PD (*n* = 1)	*SNCA* triplication	Neurons	Increased levels of α-synuclein	[38] Devine et al., 2011
PD (*n* = 2)	*SNCA* ^A53T^	Neurons	α-synuclein accumulation, axonal neuropathological changes and synaptic abnormalities	[39] Kouroupi et al., 2017
PD (*n* = 2)	*PRKN* deletion	DA neurons	Increased oxidative stress and defects in dopamine utilization	[41] Jiang et al., 2012
PD (*n* = 2)	*PRKN* deletion	Neurons	Increased oxidative stress and α-synuclein accumulation	[42] Imaizumi et al., 2012
PD (*n* = 2)	*PRKN* deletion	DA neurons	Abnormalities in mitophagy	[43] Suzuki et al., 2017
PD (*n* = 2)	*PRKN* deletion	DA neurons	Increased apoptotic tendencies and susceptibility to mitochondrial stress	[44] Tabata et al., 2018
PD (*n* = 1)	*PINK1* ^Q456X^	Neurons	Oxidative stress and mitochondrial dysfunction	[35] Cooper et al., 2012
PD (*n* = 1)	*PINK1* ^Q456X^	DA neurons	Impairment of parkin translocation to mitochondria	[40] Seibler et al., 2011
PD (*n* = 7)	Idiopathic	DA neurons	Morphological changes in neurites and impairment of autophagic clearance	[34] Sánchez-Danés et al., 2011
MSA-P & MSA-C (*n* = 6 & 4)	-	Oligodendrocytes	Producing α-synuclein during maturation	[46] Djelloul et al., 2015
MSA-P & MSA-C (*n* = 2 & 2)	-	DA neurons	Impairment of autophagic flow	[48] Monzio Compagnoni et al., 2018
FTDP-17 (*n* = 2)	*MAPT* ^N279K^	Neural stem cells	Impairment of intracellular vesicle trafficking	[49] Wren et al., 2015
FTDP-17 (*n* = 1)	*MAPT* ^N279K^	DA neurons & GABAergic neurons	Increased levels of tau fragmentation and phosphorylation	[50] Ehrlich et al., 2015
FTDP-17 (*n* = 1)	*MAPT* ^V337M^	DA neurons & GABAergic neurons	Increased levels of tau fragmentation and phosphorylation	[50] Ehrlich et al., 2015
FTDP-17 (*n* = 1)	*MAPT* ^N279K^	Cortical neurons	Altered tau expression and defects in mitochondrial transport	[51] Iovino et al., 2015
FTDP-17 (*n* = 2)	*MAPT* ^P301L^	Cortical neurons	Altered tau expression and defects in mitochondrial transport	[51] Iovino et al., 2015
FTDP-17 (*n* = 1)	*MAPT* ^IVS10+14C>T^	Cortical neurons	Accumulation of misfolded tau and extracellular release	[52] Imamura et al., 2016
FTDP-17 (*n* = 1)	*MAPT* ^R406W^	Cortical neurons	Accumulation of misfolded tau and extracellular release	[52] Imamura et al., 2016
FTDP-17 (*n* = 1)	*MAPT* ^N279K^	Astrocytes	Increased susceptibility to oxidative stress and 4R-tau expression	[53] Hallmann et al., 2017
FTDP-17 (*n* = 2)	*GRN* ^S116X^	Neurons /Microglia	Progranulin haploinsufficiency and increased susceptibility to cellular stress	[56] Almeida et al., 2012
FTDP-17 (*n* = 3)	*GRN* ^IVS1+5G>C^	Cortical neurons /Motor neurons	Progranulin haploinsufficiency and defective corticogenesis	[57] Raitano et al., 2015
Perry syndrome (*n* = 1)	*DCTN1* ^F52L^	TH-positive neurons	Dynactin aggregates in the cytoplasm	[61] Mishima et al., 2016

## References

[1] Nalls M.A., Pankratz N., Lill C.M., Do C.B., Hernandez D.G., Saad M., DeStefano A.L., Kara E., Bras J., Sharma M. (2014). Large-scale meta-analysis of genome-wide association data identifies six new risk loci for Parkinson’s disease. Nat. Genet..

[2] Chang D., Nalls M.A., Hallgrímsdóttir I.B., Hunkapiller J., van der Brug M., Cai F., Kerchner G.A., Ayalon G., International Parkinson’s Disease Genomics Consortium, 23andMe Research Team (2017). A meta-analysis of genome-wide association studies identifies 17 new Parkinson’s disease risk loci. Nat. Genet..

[3] Uryu K., Nakashima-Yasuda H., Forman M.S., Kwong L.K., Clark C.M., Grossman M., Miller B.L., Kretzschmar H.A., Lee V.M., Trojanowski J.Q. (2008). Concomitant TAR-DNA-binding protein 43 pathology is present in Alzheimer disease and corticobasal degeneration but not in other tauopathies. J. Neuropathol. Exp. Neurol..

[4] Nakashima-Yasuda H., Uryu K., Robinson J., Xie S.X., Hurtig H., Duda J.E., Arnold S.E., Siderowf A., Grossman M., Leverenz J.B. (2007). Co-morbidity of TDP-43 proteinopathy in Lewy body related diseases. Acta Neuropathol..

[5] Yokota O., Davidson Y., Bigio E.H., Ishizu H., Terada S., Arai T., Hasegawa M., Akiyama H., Sikkink S., Pickering-Brown S. (2010). Phosphorylated TDP-43 pathology and hippocampal sclerosis in progressive supranuclear palsy. Acta Neuropathol..

[6] Kouri N., Oshima K., Takahashi M., Murray M.E., Ahmed Z., Parisi J.E., Yen S.H., Dickson D.W. (2013). Corticobasal degeneration with olivopontocerebellar atrophy and TDP-43 pathology: An unusual clinicopathologic variant of CBD. Acta Neuropathol..

[7] Geser F., Malunda J.A., Hurtig H.I., Duda J.E., Wenning G.K., Gilman S., Low P.A., Lee V.M., Trojanowski J.Q. (2011). TDP-43 pathology occurs infrequently in multiple system atrophy. Neuropathol. Appl. Neurobiol..

[8] Koga S., Sanchez-Contreras M., Josephs K.A., Uitti R.J., Graff-Radford N., van Gerpen J.A., Cheshire W.P., Wszolek Z.K., Rademakers R., Dickson D.W. (2017). Distribution and characteristics of transactive response DNA binding protein 43 kDa pathology in progressive supranuclear palsy. Mov. Disord..

[9] Mishima T., Koga S., Lin W.L., Kasanuki K., Castanedes-Casey M., Wszolek Z.K., Oh S.J., Tsuboi Y., Dickson D.W. (2017). Perry syndrome: A distinctive type of TDP-43 proteinopathy. J. Neuropathol. Exp. Neurol..

[10] Schneider J.A., Arvanitakis Z., Bang W., Bennett D.A. (2007). Mixed brain pathologies account for most dementia cases in community-dwelling older persons. Neurology.

[11] Wszolek Z.K., Tsuboi Y., Ghetti B., Pickering-Brown S., Baba Y., Cheshire W.P. (2006). Frontotemporal dementia and parkinsonism linked to chromosome 17 (FTDP-17). Orphanet J. Rare Dis..

[12] Siuda J., Fujioka S., Wszolek Z.K. (2014). Parkinsonian syndrome in familial frontotemporal dementia. Park. Relat. Disord..

[13] McCann H., Stevens C.H., Cartwright H., Halliday G.M. (2014). α-Synucleinopathy phenotypes. Parkinsonism. Relat. Disord..

[14] Dickson D.W. (2018). Neuropathology of Parkinson disease. Park. Relat. Disord..

[15] Inoue H., Yamanaka S. (2011). The use of induced pluripotent stem cells in drug development. Clin. Pharmacol. Ther..

[16] Shi Y., Inoue H., Wu J.C., Yamanaka S. (2017). Induced pluripotent stem cell technology: A decade of progress. Nat. Rev. Drug Discov..

[17] Wichterle H., Przedborski S. (2010). What can pluripotent stem cells teach us about neurodegenerative diseases?. Nat. Neurosci..

[18] Jakel R.J., Schneider B.L., Svendsen C.N. (2004). Using human neural stem cells to model neurological disease. Nat. Rev. Genet..

[19] Dawson T.M., Ko H.S., Dawson V.L. (2010). Genetic animal models of Parkinson’s disease. Neuron.

[20] Jiang P., Disckson D.W. (2018). Parkinson’s disease: Experimental models and reality. Acta Neuropathol..

[21] Takahashi K., Yamanaka S. (2006). Induction of pluripotent stem cells from mouse embryonic and adult fibroblast cultures by defined factors. Cell.

[22] Takahashi K., Yamanaka S. (2007). Induction of pluripotent stem cells from adult human fibroblasts by defined factors. Cell.

[23] Seki T., Fukuda K. (2015). Methods of induced pluripotent stem cells for clinical application. World J. Stem Cells.

[24] Mazzulli J.R., Xu Y.H., Sun Y., Knight A.L., McLean P.J., Caldwell G.A., Sidransky E., Grabowski G.A., Krainc D. (2011). Gaucher disease glucocerebrosidase and α-synuclein form a bidirectional pathogenic loop in synucleinopathies. Cell.

[25] Park I.H., Arora N., Huo H., Maherali N., Ahfeldt T., Shimamura A., Lensch M.W., Cowan C., Hochedlinger K., Daley G.Q. (2008). Disease-specific induced pluripotent stem cells. Cell.

[26] Dimos J.T., Rodolfa K.T., Niakan K.K., Weisenthal L.M., Mitsumoto H., Chung W., Croft G.F., Saphier G., Leibel R., Goland R. (2008). Induced pluripotent stem cells generated from patients with ALS can be differentiated into motor neurons. Science.

[27] Ebert A.D., Yu J., Rose F.F., Mattis V.B., Lorson C.L., Thomson J.A., Svendsen C.N. (2009). Induced pluripotent stem cells from a spinal muscular atrophy patient. Nature.

[28] Soldner F., Hockemeyer D., Beard C., Gao Q., Bell G.W., Cook E.G., Hargus G., Blak A., Cooper O., Mitalipova M. (2009). Parkinson’s disease patient-derived induced pluripotent stem cells free of viral reprogramming factors. Cell.

[29] Pellegrini C., Antonioli L., Colucci R., Ballabeni V., Barocelli E., Bernardini N., Blandizzi C., Fornai M. (2015). Gastric motor dysfunctions in Parkinson’s disease: Current pre-clinical evidence. Park. Relat. Disord..

[30] Lange A., Lozano A. (1998). Parkinson’s disease: Second of two parts. N. Engl. J. Med..

[31] Mishima T., Fukae J., Fujioka S., Inoue K., Tsuboi Y. (2017). The prevalence of constipation and irritable bowel syndrome in Parkinson’s disease patients according to Rome III diagnostic criteria. J. Park. Dis..

[32] Lesage S., Brice A. (2009). Parkinson’s disease: From monogenic forms to genetic susceptibility factors. Hum. Mol. Genet..

[33] Nguyen H.N., Byers B., Cord B., Shcheglovitov A., Byrne J., Gujar P., Kee K., Schüle B., Dolmetsch R.E., Langston W. (2011). LRRK2 mutant iPSC-derived DA neurons demonstrate increased susceptibility to oxidative stress. Cell Stem Cell.

[34] Sánchez-Danés A., Richaud-Patin Y., Carballo-Carbajal I., Jiménez-Delgado S., Caig C., Mora S., di Guglielmo C., Ezquerra M., Patel B., Giralt A. (2012). Disease-specific phenotypes in dopamine neurons from human iPS-based models of genetic and sporadic Parkinson’s disease. EMBO Mol. Med..

[35] Cooper O., Seo H., Andrabi S., Guardia-Laguarta C., Graziotto J., Sundberg M., McLean J.R., Carrillo-Reid L., Xie Z., Osborn T. (2012). Pharmacological rescue of mitochondrial deficits in iPSC-derived neural cells from patients with familial Parkinson’s disease. Sci. Transl. Med..

[36] Ohta E., Nihira T., Uchino A., Imaizumi Y., Okada Y., Akamatsu W., Takahashi K., Hayakawa H., Nagai M., Ohyama M. (2015). I2020T mutant LRRK2 iPSC-derived neurons in the Sagamihara family exhibit increased Tau phosphorylation through the AKT/GSK-3β signaling pathway. Hum. Mol. Genet..

[37] Reinhardt P., Schmid B., Burbulla L.F., Schöndorf D.C., Wagner L., Glatza M., Höing S., Hargus G., Heck S.A., Dhingra A. (2013). Genetic correction of a LRRK2 mutation in human iPSCs links parkinsonian neurodegeneration to ERK-dependent changes in gene expression. Cell Stem Cell.

[38] Devine M.J., Ryten M., Vodicka P., Thomson A.J., Burdon T., Houlden H., Cavaleri F., Nagano M., Drummond N.J., Taanman J.W. (2011). Parkinson’s disease induced pluripotent stem cells with triplication of the α-synuclein locus. Nat. Commun..

[39] Kouroupi G., Taoufik E., Vlachos I.S., Tsioras K., Antoniou N., Papastefanaki F., Chroni-Tzartou D., Wrasidlo W., Bohl D., Stellas D. (2017). Defective synaptic connectivity and axonal neurophathology in a human iPSC-based model familial Parkinson’s disease. Proc. Natl. Acad. Sci. USA.

[40] Seibler P., Graziotto J., Jeong H., Simunovic F., Klein C., Krainc D. (2011). Mitochondrial Parkin recruitment is impaired in neurons derived from mutant PINK1 induced pluripotent stem cells. J. Neurosci..

[41] Jiang H., Ren Y., Yuen E.Y., Zhong P., Ghaedi M., Hu Z., Azabdaftari G., Nakaso K., Yan Z., Feng J. (2012). Parkin controls dopamine utilization in human midbrain dopaminergic neurons derived from induced pluripotent stem cells. Nat. Commun..

[42] Imaizumi Y., Okada Y., Akamatsu W., Koike M., Kuzumaki N., Hayakawa H., Nihira T., Kobayashi T., Ohyama M., Sato S. (2012). Mitochondrial dysfunction associated with increased oxidative stress and α-synuclein accumulation in PARK2 iPSC-derived neurons and postmortem brain tissue. Mol. Brain.

[43] Suzuki S., Akamatsu W., Kisa F., Sone T., Ishikawa K., Kuzumaki N., Katayama H., Miyawaki A., Hattori N., Okano H. (2017). Efficient induction of dopaminergic neuron differentiation from induced pluripotent stem cells reveals impaired mitophagy in PARK2 neurons. Biochem. Biophys. Res. Commun..

[44] Tabata Y., Imaizumi Y., Sugawara M., Andoh-Noda T., Banno S., Chai M., Sone T., Yamazaki K., Ito M., Tsukahara K. (2018). T-type calcium channels determine the vulnerability of dopaminergic neurons to mitochondrial stress in familial Parkinson disease. Stem Cell Rep..

[45] Son M.Y., Sim H., Son Y.S., Jung K.B., Lee M.O., Oh J.H., Chung S.K., Jung C.R., Kim J. (2017). Distinctive genomic signature of neural and intestinal organoids from familial Parkinson’s disease patient-derived induced pluripotent stem cells. Neuropathol. Appl. Neurobiol..

[46] Djelloul M., Holmqvist S., Boza-Serrano A., Azevedo C., Yeung M.S., Goldwurm S., Frisén J., Deierborg T., Roybon L. (2015). α-synuclein expression in the oligodendrocyte lineage: An in vitro and in vivo study using rodent and human models. Stem Cell Rep..

[47] Holmqvist S., Lehtonen Š., Chumarina M., Puttonen K.A., Azevedo C., Lebedeva O., Ruponen M., Oksanen M., Djelloul M., Collin A. (2016). Creation of a library of induced pluripotent stem cells from Parkinsonian patients. NPJ Parkinson’s Dis..

[48] Monzio Compagnoni G., Kleiner G., Samarani M., Aureli M., Faustini G., Bellucci A., Ronchi D., Bordoni A., Garbellini M., Salani S. (2018). Mitochondrial dysregulation and impaired autophagy in iPSC-derived dopaminergic neurons of multiple system atrophy. Stem Cell Rep..

[49] Wren M.C., Zhao J., Liu C.C., Murray M.E., Atagi Y., Davis M.D., Fu Y., Okano H.J., Ogaki K., Strongosky A.J. (2015). Frontotemporal dementia-associated N279K tau mutant disrupts subcellular vesicle trafficking and induces cellular stress in iPSC-derived neural stem cells. Mol. Neurodegener..

[50] Ehrlich M., Hallmann A.L., Reinhardt P., Araúzo-Bravo M.J., Korr S., Röpke A., Psathaki O.E., Ehling P., Meuth S.G., Oblak A.L. (2015). Distinct neurodegenerative changes in an induced pluripotent stem cell model of frontotemporal dementia linked to mutant TAU protein. Stem Cell Rep..

[51] Iovino M., Agathou S., González-Rueda A., Del Castillo Velasco-Herrera M., Borroni B., Alberici A., Lynch T., O’Dowd S., Geti I., Gaffney D. (2015). Early maturation and distinct tau pathology in induced pluripotent stem cell-derived neurons from patients with *MAPT* mutations. Brain.

[52] Imamura K., Sahara N., Kanaan N.M., Tsukita K., Kondo T., Kutoku Y., Ohsawa Y., Sunada Y., Kawakami K., Hotta A. (2016). Calcium dysregulation contributes to neurodegeneration in FTLD patient iPSC-derived neurons. Sci. Rep..

[53] Hallmann A.L., Araúzo-Bravo M.J., Mavrommatis L., Ehrlich M., Röpke A., Brockhaus J., Missler M., Sterneckert J., Schöler H.R., Kuhlmann T. (2017). Astrocyte pathology in a human neural stem cell model of frontotemporal dementia caused by mutant TAU protein. Sci. Rep..

[54] Verheyen A., Diels A., Reumers J., van Hoorde K., van den Wyngaert I., van Outryve d’Ydewalle C., de Bondt A., Kuijlaars J., de Muynck L., de Hoogt R. (2018). Genetically engineered iPSC-derived FTDP-17 MAPT neurons display mutation-specific neurodegenerative and neurodevelopmental phenotypes. Stem Cell Rep..

[55] Nguyen A.D., Nguyen T.A., Zhang J., Devireddy S., Zhou P., Karydas A.M., Xu X., Miller B.L., Rigo F., Ferguson S.M. (2018). Murine knockin model for progranulin-deficient frontotemporal dementia with nonsense-mediated mRNA decay. Proc. Natl. Acad. Sci. USA.

[56] Almeida S., Zhang Z., Coppola G., Mao W., Futai K., Karydas A., Geschwind M.D., Tartaglia M.C., Gao F., Gianni D. (2012). Induced pluripotent stem cell models of progranulin-deficient frontotemporal dementia uncover specific reversible neuronal defects. Cell Rep..

[57] Raitano S., Ordovàs L., de Muynck L., Guo W., Espuny-Camacho I., Geraerts M., Khurana S., Vanuytsel K., Tóth B.I., Voets T. (2015). Restoration of progranulin expression rescues cortical neuron generation in an induced pluripotent stem cell model of frontotemporal dementia. Stem Cell Rep..

[58] Mishima T., Fujioka S., Tomiyama H., Yabe I., Kurisaki R., Fujii N., Neshige R., Ross O.A., Farrer M.J., Dickson D.W. (2017). Establishing diagnostic criteria for Perry syndrome. J. Neurol. Neurosurg. Psychiatry.

[59] Farrer M.J., Hulihan M.M., Kachergus J.M., Dächsel J.C., Stoessl A.J., Grantier L.L., Calne S., Calne D.B., Lechevalier B., Chapon F. (2009). DCTN1 mutations in Perry syndrome. Nat. Genet..

[60] Mishima T., Deshimaru M., Watanabe T., Kubota K., Kinoshita-Kawada M., Yuasa-Kawada J., Takasaki K., Uehara Y., Jinno S., Iwasaki K. (2018). Behavioral defects in a DCTN1^G71A^ transgenic mouse model of Perry syndrome. Neurosci. Lett..

[61] Mishima T., Ishikawa T., Imamura K., Kondo T., Koshiba Y., Takahashi R., Takahashi J., Watanabe A., Fujii N., Tsuboi Y. (2016). Cytoplasmic aggregates of dynactin in iPSC-derived tyrosine hydroxylase-positive neurons from a patient with Perry syndrome. Park. Relat. Disord..

[62] Hosaka Y., Inoshita T., Shiba-Fukushima K., Cui C., Arano T., Imai Y., Hattori N. (2017). Reduced TDP-43 expression improves neuronal activities in a drosophila model of Perry syndrome. EBioMedicine.

[63] Alami N.H., Smith R.B., Carrasco M.A., Williams L.A., Winborn C.S., Han S.S.W., Kiskinis E., Winborn B., Freibaum B.D., Kanagaraj A. (2014). Axonal transport of TDP-43 mRNA granules in neurons is impaired by ALS-causing mutations. Neuron.

[64] Maury Y., Côme J., Piskorowski R.A., Salah-Mohellibi N., Chevaleyre V., Peschanski M., Martinat C., Nedelec S. (2015). Combinatorial analysis of developmental cues efficiently converts human pluripotent stem cells into multiple neuronal subtypes. Nat. Biotechnol..

[65] Paull D., Sevilla A., Zhou H., Hahn A.K., Kim H., Napolitano C., Tsankov A., Shang L., Krumholz K., Jagadeesan P. (2015). Automated, high-throughput derivation, characterization and differentiation of induced pluripotent stem cells. Nat. Methods.

[66] Björklund A. (1993). Better cells for brain repair. Nature.

[67] Barker R.A., Drouin-Ouellet J., Parmar M. (2015). Cell–based therapies for Parkinson disease—Past insights and future potential. Nat. Rev. Neurol..

[68] Barker R.A., Barrett J., Mason S.L., Björklund A.F. (2013). Fetal dopaminergic transplantation trials and the future of neural grafting in Parkinson’s disease. Lancet Neurol..

[69] Doi D., Morizane A., Kikuchi T., Onoe H., Hayashi T., Kawasaki T., Motono M., Sasai Y., Saiki H., Gomi M. (2012). Prolonged maturation culture favors a reduction in the tumorigenicity and the dopaminergic function of human ESC-derived neural cells in a primate model of Parkinson’s disease. Stem Cells.

[70] Brederlau A., Correia A.S., Anisimov S.V., Elmi M., Paul G., Roybon L., Morizane A., Bergquist F., Riebe I., Nannmark U. (2006). Transplantation of human embryonic stem cell-derived cells to a rat model of Parkinson’s disease: Effect of in vitro differentiation on graft survival and teratoma formation. Stem Cells.

[71] Doi D., Samata B., Katsukawa M., Kikuchi T., Morizane A., Ono Y., Sekiguchi K., Nakagawa M., Parmar M., Takahashi J. (2014). Isolation of human induced pluripotent stem cell-derived dopaminergic progenitors by cell sorting for successful transplantation. Stem Cell Rep..

[72] Samata B., Doi D., Nishimura K., Kikuchi T., Watanabe A., Sakamoto Y., Kakuta J., Ono Y., Takahashi J. (2016). Purification of functional human ES and iPSC-derived midbrain dopaminergic progenitors using LRTM1. Nat. Commun..

[73] Bye C.R., Jönsson M.E., Björklund A., Parish C.L., Thompson L.H. (2015). Transcriptome analysis reveals transmembrane targets on transplantable midbrain dopamine progenitors. Proc. Natl. Acad. Sci. USA.

[74] Kikuchi T., Morizane A., Doi D., Magotani H., Onoe H., Hayashi T., Mizuma H., Takara S., Takahashi R., Inoue H. (2017). Human iPS cell-derived dopaminergic neurons function in a primate Parkinson’s disease model. Nature.

[75] Lee P.H., Kim J.W., Bang O.Y., Ahn Y.H., Joo I.S., Huh K. (2008). Autologous mesenchymal stem cell therapy delays the progression of neurological deficits in patients with multiple system atrophy. Clin. Pharmacol. Ther..

[76] Woodruff G., Young J.E., Martinez F.J., Buen F., Gore A., Kinaga J., Li Z., Yuan S.H., Zhang K., Goldstein L.S. (2013). The presenilin-1 ΔE9 mutation results in reduced γ-secretase activity, but not total loss of PS1 function, in isogenic human stem cells. Cell Rep..

[77] Charpentier E., Doudna J.A. (2013). Biotechnology: Rewriting a genome. Nature..

[78] Hsu P.D., Lander E.S., Zhang F. (2014). Development and applications of CRISPR-Cas9 for genome engineering. Cell.

[79] Yusa K., Rashid S.T., Strick-Marchand H., Varela I., Liu P.-Q., Paschon D.E., Miranda E., Ordóñez A., Hannan N.R., Rouhani F.J. (2011). Targeted gene correction of α1-antitrypsin deficiency in induced pluripotent stem cells. Nature.

[80] Paquet D., Kwart D., Chen A., Sproul A., Jacob S., Teo S., Olsen K.M., Gregg A., Noggle S., Tessier-Lavigne M. (2016). Efficient introduction of specific homozygous and heterozygous mutations using CRISPR/Cas9. Nature.

[81] Slaymaker I.M., Gao L., Zetsche B., Scott D.A., Yan W.X., Zhang F. (2016). Rationally engineered Cas9 nucleases with improved specificity. Science.

[82] Howden S.E., McColl B., Glaser A., Vadolas J., Petrou S., Little M.H., Elefanty A.G., Stanley E.G. (2016). A Cas9 Variant for Efficient Generation of Indel-Free Knockin or Gene-Corrected Human Pluripotent Stem Cells. Stem Cell Rep..

[83] Poon A., Zhang Y., Chandrasekaran A., Phanthong P., Schmid B., Nielsen T.T., Freude K.K. (2017). Modeling neurodegenerative diseases with patient-derived induced pluripotent cells: Possibilities and challenges. New Biotechnol..

[84] Pașca S.P. (2018). The rise of three-dimensional human brain cultures. Nature.

[85] Paşca A.M., Sloan S.A., Clarke L.E., Tian Y., Makinson C.D., Huber N., Kim C.H., Park J.Y., O’Rourke N.A., Nguyen K.D. (2015). Functional cortical neurons and astrocytes from human pluripotent stem cells in 3D culture. Nat. Methods.

[86] Jo J., Xiao Y., Sun A.X., Cukuroglu E., Tran H.D., Göke J., Tan Z.Y., Saw T.Y., Tan C.P., Lokman H. (2016). Midbrain-like Organoids from Human pluripotent stem cells contain functional dopaminergic and neuromelanin-producing neurons. Cell Stem Cell.

[87] Kalia L.V., Lang A.E. (2015). Parkinson’s disease. Lancet.

[88] Sidransky E., Nalls M.A., Aasly J.O., Aharon-Peretz J., Annesi G., Barbosa E.R., Bar-Shira A., Berg D., Bras J., Brice A. (2009). Multicenter analysis of glucocerebrosidase mutations in Parkinson’s disease. N. Engl. J. Med..

[89] Wang W., Wang X., Fujioka H., Hoppel C., Whone A.L., Caldwell M.A., Cullen P.J., Liu J., Zhu X. (2016). Parkinson’s disease-associated mutant VPS35 causes mitochondrial dysfunction by recycling DLP1 complexes. Nat. Med..

[90] Burbulla L.F., Song P., Mazzulli J.R., Zampese E., Wong Y.C., Jeon S., Santos D.P., Blanz J., Obermaier C.D., Strojny C. (2017). Dopamine oxidation mediates mitochondrial and lysosomal dysfunction in Parkinson’s disease. Science.

[91] Studer L., Vera E., Cornacchia D. (2015). Programming and reprogramming cellular age in the era of induced pluripotency. Cell Stem Cell.

[92] Miller J.D., Ganat Y.M., Kishinevsky S., Bowman R.L., Liu B., Tu E.Y., Mandal P., Vera E., Shim J.-W., Kriks S. (2013). Human iPSC-based modeling of late-onset disease via progerin-induced aging. Cell Stem Cell.

[93] Compagnucci C., Bertini E. (2017). The potential of iPSCs for the treatment of premature aging disorders. Int. J. Mol. Sci..

